# Comparison of branched, fenestrated, and parallel strategies for endovascular treatment of thoracoabdominal aortic pathologies involving visceral regions

**DOI:** 10.3389/fcvm.2024.1416635

**Published:** 2024-09-24

**Authors:** Xiaochen Ma, Zhishi Wu, Guanglang Zhu, Mingjin Guo, Yongxin Li, Junjun Liu, Mingyuan Liu, Youjin Li, Bo Ye, Tao Chen, Ming Qi, Hongyan Xiao, Zhaoxiang Zeng, Yudong Sun, Rui Feng, Zaiping Jing, Jiaxuan Feng

**Affiliations:** ^1^ Department of Vascular Surgery, Changhai Hospital, Navy Medical University, Shanghai, China; ^2^ Department of Vascular Surgery, Ruijin Hospital, Affiliated to Medical School of Shanghai Jiaotong University, Shanghai, China; ^3^ Department of Vascular Surgery, Affiliated Hospital of Qingdao University, Qingdao University, Shandong, China; ^4^ Department of Vascular Surgery, Beijing Friendship Hospital, Capital Medical University, Beijing, China; ^5^ Department of Cardiovascular Surgery, People’s Hospital of Ningxia Hui Autonomous Region, Yinchuan, China; ^6^ The Department of Vascular & Hernia Surgery, Ganzhou People’s Hospital, Ganzhou, Jiangxi, China; ^7^ Department of Vascular Surgery, Wuhan Asia Heart Hospital, Wuhan, Hubei, China; ^8^ Department of Vascular Surgery, Shanghai General Hospital, Shanghai Jiao Tong University School of Medicine, Shanghai, China; ^9^ Department of Vascular Surgery, Shanghai Fourth People’s Hospital, School of Medicine, Tongji University, Shanghai, China

**Keywords:** aortic aneurysm, aortic dissection, endovascular repair, visceral arteries, branched stent graft, parallel stent graft, fenestrated stent graft

## Abstract

**Purpose:**

To compare the long-term efficacy of the parallel stent graft (PSG), fenestrated stent graft (FSG), and branched stent graft (BSG) techniques to treat thoracoabdominal aortic pathologies.

**Materials and methods:**

In total, 291 patients with thoracic aortic aneurysm (TAA) and dissection (TAD) involving visceral arteries who underwent PSG (*n* = 85; 15 TAA and 70 TAD), FSG (*n* = 107; 47 TAD and 60 TAA), or BSG (*n* = 99; 37 TAD and 62 TAA) were included from multiple centers from January 2015 to December 2022, and a total of 1,108 visceral aortic branches were reconstructed.

**Results:**

The average reconstruction time of each visceral aortic branch for FSG, BSG, and PSG is 27.5 ± 12.1, 23.2 ± 11.9, and 18.8 ± 11.8 min, respectively (*P* < 0.01). The free-from-endoleak rate at the last follow-up for FSG, BSG, and PSG was 86.9%, 91.9%, and 60.0%, respectively. The last follow-up patency rate for FSG, BSG, and PSG was 85.0%, 91.9%, and 94.1%, respectively. The average reconstruction price of each visceral aortic branch for FSG, BSG, and PSG was 41.40 ± 3.22 thousand RMB, 41.84 ± 3.86 thousand RMB, and 42.35 ± 4.52 thousand RMB, respectively (*P* = 0.24).

**Conclusion:**

To treat the aortic pathologies involving the visceral segment, BSG had a lower endoleak rate and higher branch patency rate when compared with the FSG and PSG techniques. The expense of BSG was comparable to the other two techniques.

## 1 Introduction

Thoracic and abdominal aortic pathologies involving abdominal visceral areas include thoracic and abdominal aortic aneurysm, visceral aortic pseudoaneurysm, and aortic dissection aneurysm with distal false lumen. For example, the incidence of aortic dissection aneurysm with distal false lumen is not low, especially in the follow-up period after type A dissection surgery (1).

Thoracic and abdominal aortic pathologies can be treated with open surgery, hybrid surgery, or endovascular therapy. Open repair remains the first-line therapy, particularly in patients with connective tissue disorders. However, these procedures are associated with high morbidity and mortality rates, even in centers of excellence. Open arch repairs after acute type A surgery are associated with a 9% 30-day mortality and a 15% stroke rate. Open repair of postdissection thoracic aortic aneurysm (TAA) is associated with early mortality (8.3%), spinal cord ischemia (SCI; 1.3%), stroke (2.9%), and dialysis (5%) risks. The early reported experience of the endovascular management of postdissection TAA is encouraging and has been associated with favorable early outcomes (2). Endovascular aneurysm repair proved to have lower early morbidity and mortality compared with open repair in prospective randomized trials (3). There are three main options for endovascular therapy: parallel stent graft (PSG), fenestrated stent graft (FSG), and branched stent graft (BSG) techniques. However, endovascular treatment remained a challenge because of the need to reconstruct the visceral branch arteries. Endovascular strategies such as PSG, FSG, and BSG techniques have been reported for years (2–6). There are studies that have reported results for respective strategies, but the comparison among the three strategies in thoracoabdominal aortic pathologies is still lacking.

To compare the long-term results of the three endovascular strategies, data from multiple centers were collected and analyzed. The long-term results, safety, and economic cost were compared among the three groups.

## 2 Materials and methods

### 2.1 Patient enrollment

This was a multicenter retrospective study from 10 centers. From January 2015 to December 2022, there were 291 patients with thoracoabdominal aortic aneurysm and dissection involving visceral regions who underwent PSG [*n* = 85, 15 TAA and 70 thoracic aortic dissection (TAD)], FSG (*n* = 107, 60 TAA and 47 TAD), and BSG (*n* = 99, 62 TAA and 37 TAD). A total of 1,108 visceral aortic branches (*N* = 1,108) were reconstructed. This study was approved by the ethics committee and obtained informed consent from all patients.

The inclusion criteria were as follows: (a) aortic pathologies including aneurysm, and aortic dissection, involving abdominal visceral areas; (b) maximal diameter of aortic aneurysm ≥5.5 cm; (c) patients with aortic dissections pending rupture or aortic growth of >1 cm per year or >0.5 cm per half a year; (d) received endovascular repair (EVAR).

**Figure 1:**
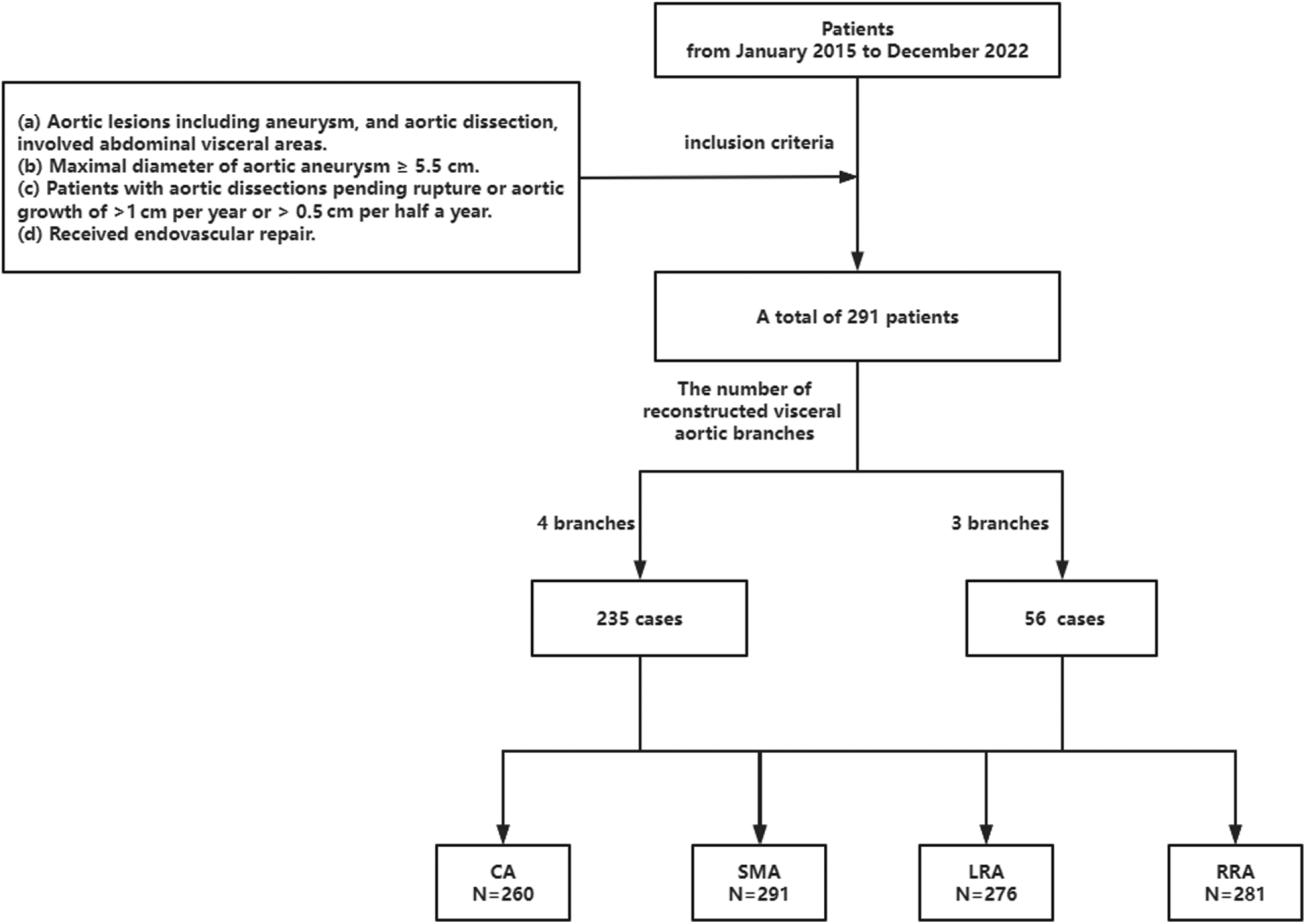
Screening process. CA, celiac axis; SMA, superior mesenteric artery; LRA, left renal artery; RRA, right renal artery.

The exclusion criteria were as follows: (a) open surgery and hybrid surgery; (b) the combination of two or three kinds of PSG, FSG, and BSG; (c) aortic pseudoaneurysm involving visceral arteries, which might be caused by mycotic or inflammatory lesions; (d) patients who were unable to provide informed consent or participate in long-term follow-up. Figure 1 provides an overview of the patient enrollment process.

### 2.2 Surgical procedures

#### 2.2.1 FSG

All patients were provided general anesthesia in the supine position. Their left brachial artery and bilateral femoral arteries were then exposed and the fenestration of the stent graft was customized on the table according to the preoperative computed tomography angiography (CTA) (7).

The constraint guidewire was sutured at 6 o’clock of the stent graft and then the stent graft was folded back into the delivery catheter.

After re-sheathing the FSG, the delivery catheter was introduced to the ideal location during the angiography. The stent graft was slowly deployed during angiography to make the fenestration align with the origin of the visceral arteries. The guidewire and catheter went through the fenestrations into the relevant visceral arteries one by one. Viabahn stent grafts (Gore, Newark, DE, USA) were delivered and deployed to bridge the branch arteries and the fenestrations.

#### 2.2.2 BSG

**Figure 2:**
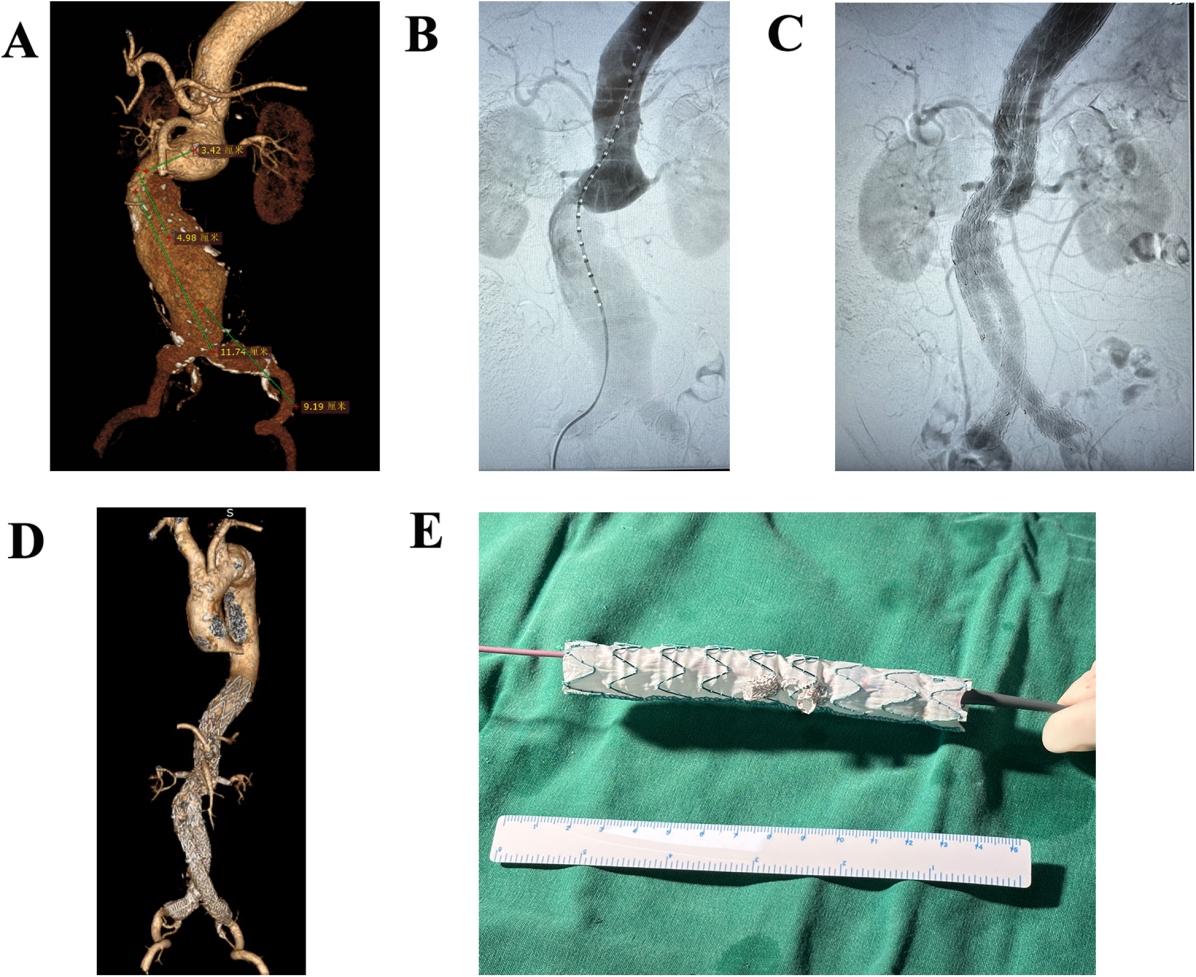
Preoperative and postoperative CTA images of a patient who underwent EVAR. **(A)** Preoperative CTA image of thoracoabdominal aortic aneurysms. **(B)** Intraoperative CTA image. **(C)** Final postoperative CTA image. **(D)** Follow-up CTA demonstrates satisfactory aortic remodeling without endoleaks. **(E)** A picture of BSG.

The procedure details of BSG have been described in previous literature (8). The mini-cuff on the stent graft was made using a Viabahn stent graft (Gore, USA). Figure 2 shows the preoperative and postoperative CTA images of a patient as an example.

#### 2.2.3 PSG

The procedure details of PSG have been described in previous literature from this team (9).

### 2.3 Measurement and follow-up

The operational difficulty of the repair was measured by the average time of reconstructing a single visceral aortic branch (T=totaltime/numberofbranches). The surgical cost was measured by the average price required for the reconstruction of a single visceral aortic branch [C=(totalcost−aorticstentgraftcost)/ numberofbranches].

The postoperative follow-up protocol for the patients included in the study involved performing a CTA every 3 months during the first year after the operation. Subsequently, the patients underwent a CTA every half a year in the second year, and then annually in the following years. The evaluation of the CTA images was conducted by two experienced vascular surgeons who were responsible for assessing the various parameters and outcomes.

The primary focus of the evaluation was to identify any adverse events, including but not limited to death, endoleak (leakage of blood outside the stent graft), stent graft migration, organ ischemia (reduced blood flow to organs), spinal cord ischemia, aneurysm enlargement, and branch graft occlusion. These events were considered important indicators of the long-term success and safety of the EVAR.

### 2.4 Statistical analysis

Continuous variables were summarized as mean ± standard deviation. Skewed variables were summarized as median and range. The Kaplan–Meier analysis was employed to compare the follow-up for free from endoleak among the three different repair techniques. The data obtained from the study were analyzed using SPSS for Windows software (version 26.0.0.0, IBM Corporation, Armonk, NY, USA). A *P*-value <0.05 was considered significant.

## 3 Results

### 3.1 Patient demographics

In the study, a total of 291 patients who met the criteria from January 2015 to December 2022 were enrolled. Among these patients, 156 were diagnosed with aortic dissection. Out of these patients, 47 underwent FSG repair, 37 received BSG, and 70 received PSG repair. The remaining 137 patients were diagnosed with aortic aneurysm. Among these patients, 60 underwent FSG repair, 62 received BSG repair, and 15 received PSG repair. In terms of the number of aortic branches reconstructed, 235 patients had all four aortic branches reconstructed, while 56 patients had only three aortic branches reconstructed.

A summary of the patient demographics is presented in Table 1. There were no statistically significant differences in average ages among the FSG (66.77 ± 10.09), BSG (64.92 ± 9.26), and PSG groups (66.98 ± 9.02) (*P*_1 _= 0.17, *P*_2 _= 0.88, *P*_3 _= 0.14, *P* = 0.26 > 0.05). Similarly, there were no statistically significant differences in sex among the three groups (male: FSG: 91.59% vs. BSG: 85.86% vs. PSG: 88.24%, *P*_1 _= 0.20, *P*_2 _= 0.47, *P*_3 _= 0.61, *P* = 0.47 > 0.05). In addition, there were no significant differences between the groups in terms of risk factors associated with vascular diseases, including smoking history (FSG: 50.47% vs. BSG: 49.49% vs. PSG: 49.41%, *P*_1 _= 0.89, *P*_2 _= 0.89, *P*_3 _= 0.99, *P* = 0.99 > 0.05), hypertension (FSG: 74.77% vs. BSG: 80.81% vs. PSG: 76.47%, *P*_1 _= 0.30, *P*_2 _= 0.78, *P*_3 _= 0.49, *P* = 0.57 > 0.05), and diabetes mellitus (FSG: 19.63% vs. BSG: 21.21% vs. PSG:29.41%, *P*_1 _= 0.79, *P*_2 _= 0.11, *P*_3 _= 0.19, *P* = 0.24 > 0.05).

**Table 1:** Summary of patient demographics.

	Fenestrated stent graft	Branched stent graft	Parallel stent graft	*P*-value
Age (years)	66.77 ± 10.09	64.92 ± 9.26	66.98 ± 9.02	0.26
Male	98 (91.59%)	85 (85.86%)	75 (88.24%)	0.43
Smoking history	54 (50.47%)	49 (49.49%)	42 (49.41%)	0.99
Hypertension	80 (74.77%)	80 (80.81%)	65 (76.47%)	0.57
Diabetes	21 (19.63%)	21 (21.21%)	25 (29.41%)	0.24

*P *= FSG vs. BSG vs. PSG.

### 3.2 In-hospital comparisons

The operational difficulty of the repair was assessed by measuring the average time required to reconstruct a single visceral artery branch. The results showed that FSG had the longest average time of 27.5 ± 12.1 min, followed by BSG with an average time of 23.2 ± 11.9 min, and PSG with the shortest average time of 18.8 ± 11.8 min (*P* < 0.01).

The surgical cost was evaluated by determining the average cost required for the reconstruction of a single visceral artery branch. The average reconstruction price of each visceral aortic branch for FSG, BSG, and PSG was 41.40 ± 3.22 thousand RMB, 41.84 ± 3.86 thousand RMB, and 42.35 ± 4.52 thousand RMB, respectively (*P* = 0.24 > 0.05). This shows that the expense of BSG was comparable to the other two techniques.

In all the included patients, there were no deaths in the three groups. The endoleakage rate discovered in final angiography during surgery for FSG, BSG, and PSG was 8.4%, 3.0%, and 29.4%, respectively. Five patients in the PSG group and two patients in the BSG group experienced stent graft migration. Furthermore, 15 patients in the FSG group, 4 patients in the PSG group, and 3 patients in the BSG group developed organ ischemia (kidney or spleen). One patient in the FSG group developed spinal cord ischemia. Four patients in the FSG group and 13 patients in the PSG group had an aneurysm enlargement due to endoleak. Finally, 21 patients in the FSG group, 7 patients in the PSG group, and 4 patients in the BSG group experienced branch graft occlusion.

### 3.3 Follow-up outcomes

**Figure 3:**
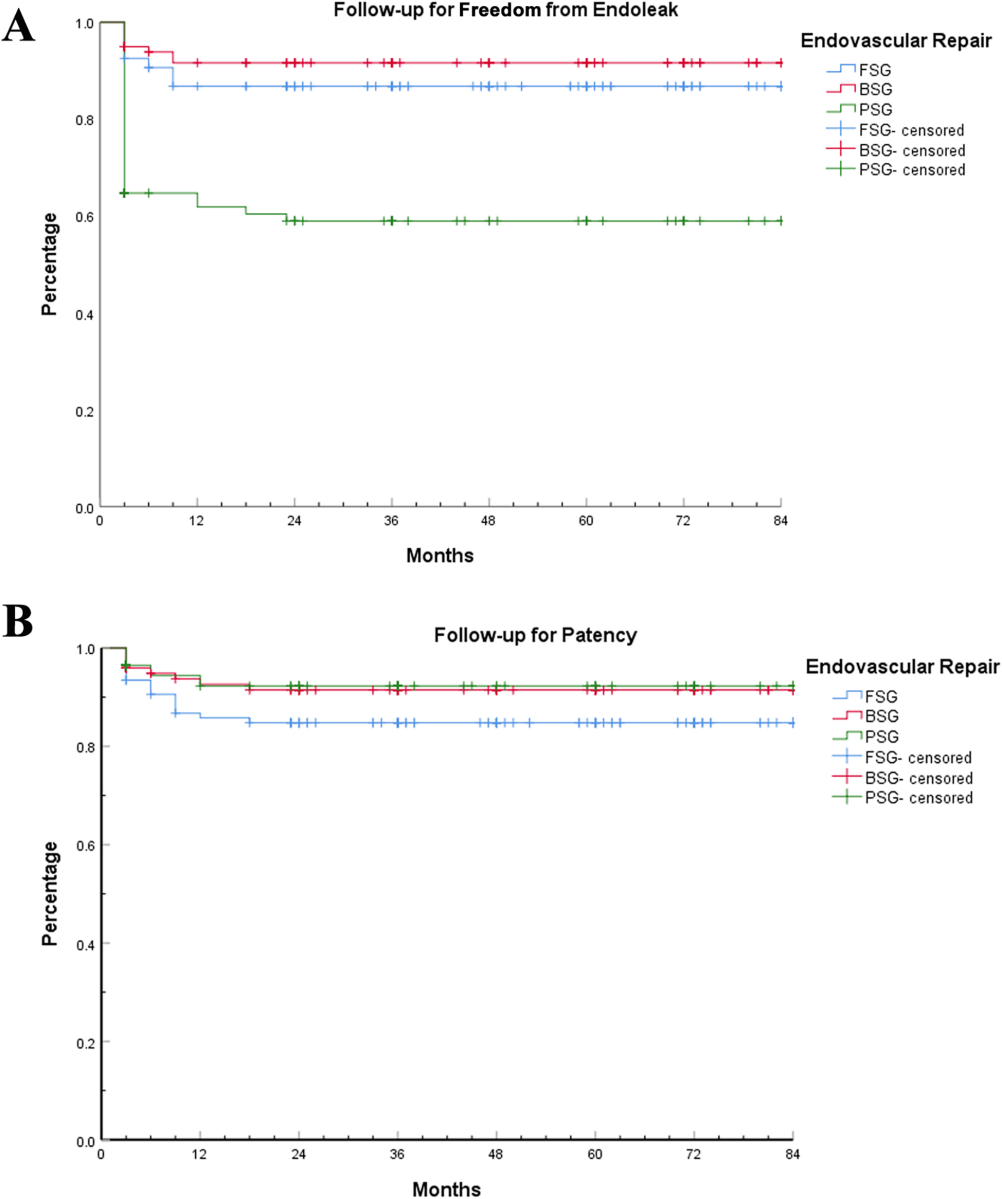
Follow-up for free-from-endoleak and patency rates. Kaplan–Meier analysis of follow-up for free-from-endoleak and patency rates among the three EVAR techniques. **(A)** Follow-up for free-from-endoleak rate. **(B)** Follow-up for patency rate.

The endoleakage rate and patency rate during the follow-up period were determined based on the last CTA. The average follow-up time for FSG, BSG, and PSG was 45.86 ± 26.32, 44.13 ± 26.71, and 43.31 ± 24.11, respectively (*P*_1 _= 0.63, *P*_2 _= 0.50, *P*_3 _= 0.83, *P* = 0.78 > 0.05). There was no difference in follow-up time among the groups. Figure 3 shows that the free-from-endoleak rate at follow-up for FSG, BSG, and PSG was 86.9%, 91.9%, and 60.0%, respectively. The follow-up patency rate for FSG, BSG, and PSG was 85.0%, 91.9%, and 94.1%, respectively. The results showed that BSG had the lowest endoleak rate and highest branch patency rate, indicating better long-term efficacy.

During the follow-up period, three patients died in the PSG group and two of these died of aortic aneurysm rupture due to endoleak after endovascular surgery. Two patients died in the FSG group and one of these died of an aortic ulcer and infection. Three patients died in the BSG group and none of these died of aortic disease or complications. As for complications during follow-up, 14 patients had an endoleak, 1 patient had an aortic ulcer and infection, and 2 patients had stent graft occlusion in the FSG group. Furthermore, 8 patients had an endoleak in the BSG group, and 34 patients had an endoleak and 2 patients had aortic aneurysm rupture in the PSG group. No patients had their endovascular surgery transform into open surgery, but four patients had an endovascular secondary intervention in the PSG group compared with no endovascular secondary interventions in the FSG and BSG groups.

## 4 Discussion

Thoracic and abdominal aortic pathologies mainly include true aneurysm, dissection, and pseudoaneurysm of the thoracic and abdominal aorta. Among them, the incidence rate of thoracic aortic aneurysm is 5–10 per 100,000 person years (10), and the incidence rate of abdominal aortic aneurysm is approximately three times that of thoracic aortic aneurysm (11). The incidence of aortic dissection is estimated to be 5–30 cases per million people per year (10). Pseudoaneurysm of the thoracic and abdominal aorta is caused by infection, trauma, and iatrogenic factors, and mostly appears as a complication of true aneurysm and intracavitary dissection treatment.

Procedures to repair proximal aortic dissection have improved the survival rate of acute phase patients tremendously. The false lumen of aortic dissection can be diminished by open repair or thrombosis and obliterated after stent graft repair and true lumen remodeling thereafter (12, 13). Nevertheless, most of the distal intimal tears and false lumen would exist consistently without intervention, especially in the distending aorta (14) and abdominal aorta (15). Most patients with Stanford type A aortic dissection would live with a patent distal false lumen after open repair of the ascending aorta with a speed of processing pathology as fast as 1–3.7 mm per year (16). The false lumen would become a dissection aneurysm eventually and threaten patients’ lives. Chronic aortic dissection is the second reason for aortic aneurysm. Approximately 9% of Stanford type A aortic dissection patients need a second intervention for distal dissection aneurysm after the proximal procedure (14). As for Stanford type B aortic dissection after thoracic endovascular repair (TEVAR), the prevalence is 7.8% (14).

There is no level I data supporting a size threshold for the repair of TAAs. The decision to treat TAAs is based on the risk of rupture and complications against the risk of morbidity and mortality due to the intervention (17). A 2010 joint guideline on thoracic aortic disease suggested open repair for TAA at a threshold of 5.5 cm for patients without comorbidities with TAAs secondary to chronic dissection and EVAR for those with degenerative or secular aneurysms (18). The background was less developed endovascular techniques. As endovascular techniques have developed and showed promise in TAA treatment, we began to treat some chronic dissection patients with complications or at a threshold of 5.5 cm. Patient selection was largely based on the high risk of morbidity and mortality of open aortic repair with 95.2% (20/21) of patients categorized as Crawford I (4.8%, 1/21), II (33.3%, 7/21), III (52.3%, 11/21), and V (4.8%, 1/21) with high risk of open repair. One Crawford IV patient was 83 years old with chronic obstructive pulmonary disease (COPD) and cardiac inefficiency and was deemed a poor candidate for open aortic repair (9).

Open surgery repair has been recognized as the first means of treating thoracic and abdominal aortic lesions and was the gold standard (19–21); however, it also causes severe complications and mortality. For example, evidence shows that perioperative mortality cases range from 5%–19% and the paraparesis rate is between 3.8% and 15.1%. Compared with open surgery, EVAR has the risk of stent graft occlusion and endoleak but it also has promising short-term and mid-term outcomes.

There are several procedures for treating aortic aneurysm. Open repair with prosthesis graft replacement of aneurysmal aorta was the first method of treating thoracic abdominal aortic aneurysm and pararenal aortic aneurysm and is also the gold standard (20–23). However, this method needs a large incision to expose the aorta, the aorta is clamped for a long time, and there is inevitable ischemia of abdominal organs. In addition, there is a much higher risk of spinal cord ischemia due to direct damage of intercostal arteries. The mortality of open surgery is as high as 7%–17% (22) and is especially high for Crawford type Ⅱ thoracic abdominal aortic aneurysm (23). In our previous study, 95.2% (20/21) of the patients involved had a descending thoracic aorta and were deemed high-risk patients for open surgery. This is a very difficult technique and can only be applied in a small number of large and experienced centers. Older patients or patients with multiple comorbidities are deemed poor candidates for this technique.

The debranching procedure was initially used to treat TAA in 1999 (24). As a hybrid procedure that combined open repair and endovascular therapy, it was less invasive, reducing organ ischemia and causing fewer respiratory system complications. However, an abdominal opening under general anesthesia is also needed which is a great challenge for senior patients or those with a hostile abdomen.

Total endovascular therapy is an alternative for patients who are deemed poor candidates for open or hybrid procedures. The branched and fenestrated EVAR techniques have shown good safety and efficiency in treating pararenal AAs and some TAAs. However, there are very strict limitations for anatomic character and patients should be stringently selected by accurate preoperative CTA examination and measurement. The origin of branch arteries must be good for fenestrated EVAR to be positioned; the space of the true lumen must be sufficient for branch EVAR to expand and select the branch arteries. But for TAD with a narrow and sometimes circuitous true lumen compressed by a false lumen and a highly variable origin of branch arteries, the branched and fenestrated EVAR techniques are not a good choice. The minimum preoperative diameter of the true lumen was 12.3 ± 4.8 mm in our study. Furthermore, the long time required for customization of a branched EVAR stent graft is a disadvantage. They also remain costly (25) and may not be fit for urgent or semi-urgent patients.

The parallel stent graft technique is defined as putting two or more stent grafts in one lumen to supply several different arteries. It is first invented to bail out the miscovered renal artery in the EVAR procedure. After modification and improvement, this technique has been widely used in treating pararenal AAs and some TAAs. There are marked advantages such as easy to maneuver, availability of materials, and flexible adaptation. Thus, it can be easily used in medical centers with basic conditions. Several large volume studies have proved that the parallel stent graft technique showed safety and efficiency in treating pararenal AAs and some TAAs (26, 27).

The patency of the false lumen and distal intimal tears are independent risk factors for chronic dilation of aortic dissection (28, 29). The intimal tear acts as an inflow and maintains the patency of the false lumen. Sealing the proximal intimal tear can lower the pressure of the false lumen and induce thrombosis and eventually remodel the true lumen. TEVAR is widely used to treat acute aortic dissection and has proven efficient (30, 31). Even uncomplicated type B aortic dissection can benefit from TEVAR (32). It is a good alternative for chronic aortic dissection, especially in patients deemed poor open repair candidates, to seal the intimal tear with a stent graft. However, the visceral district of the aorta limits the use of standard endovascular therapy because of the difficulty in reconstructing branch arteries. Parallel stent grafts can bridge the true lumen and branch artery across the intimal tear and false lumen, sealing the intimal tear at the same time as reconstructing the branch artery.

The most common complications of the parallel stent technique are endoleaks and occlusion of branch stent. In our previous study, we used coil and glue to assist in sealing the intimal tear and inducing thrombosis of the false lumen. Any type I a or Ib or type III endoleak during digital subtraction angiography (DSA) was treated with a coil and glue in our study. In total, 28.6% (6/21) patients were detected to have a type I or III endoleak during DSA. All were treated with a coil or Onyx glue embolization in the gutter. Full thrombosis of the false lumen was achieved in most cases (85.7%, 18/21). Two cases did not achieve full thrombosis of the false lumen because of a type II endoleak. There was no lesion progression observed in any patients. Those with part thrombosis of false lumen had a consistent diameter of the total aorta and showed no expansion during follow-up. The maximum diameter of the total aorta at follow-up showed a shrinking trend.

As to length of overlap between the branch stent graft and the main body, some specialists suggest as long as 5 instead of 2 cm to reduce endoleaks. In our experience, a 2–3 cm overlap is enough and no type Ⅱ endoleak was seen in the TAD. For TAD, the aims are to seal the tears and diminish the inflow and outflow of the false lumen with the branch arteries being validly reconstructed. Concepts such as a neck aneurysm and sufficient anchoring zone are not the same as a degenerative aortic aneurysm. As to the length of stenting in the branch arteries, 4–5 cm is preferred because stent grafts in a large aneurysm sac can swing and migrate with the heartbeat. If there is not enough landing zone, for example if the renal artery trunk is 2 cm only, a bare metal stent can be placed in advance to extend the anchor zone.

Acute occlusions of the branch stents are mostly due to mechanical reasons such as compression or kinks that lead to severe blood flow reduction. Our experience is to expand the balloon sufficiently and line the bare metal stent in the parallel stent graft aggressively if a kink or stenosis exists after balloon expansion. Bare stents were used (37.9%, *n* = 25) where necessary. In our previous study, a heparin banded weaved stent graft, Viabahn (Gore), was primarily chosen over (60, 90.9%) a laser-engraved cover stent such as Fluency (BARD, New Providence, NJ, USA) (6, 9.1%) for the flexibility and long enough length to avoid acute thrombosis and kinking. Furthermore, the main body of the Excluder (Gore) is much more flexible due to the continuous little wave weaved structure and can avoid compression in the parallel stent graft. One of our patients suffered from acute loss of their right kidney postoperatively. Emergency angiography confirmed thrombosis and a narrow renal stent graft. It was treated by catheter-directed thrombolysis and a bare metal stent was lined in the stenosis. One patient with occlusion of branch stent graft of renal arteries was seen in the follow-up period. There was moderate atherosclerosis in the run-off. No patient needed permanent hemodialysis though. The hyperplasia of the anastomosis may have contributed to the later restenosis and occlusion of the branch artery. We may see it in follow-up over a longer duration.

Another problem is ischemia of organs and the spinal cord due to long segment covering of the aorta. The reintervention of aortic dissection may face the challenge of covering almost all the intercostal arteries and lumbar arteries. Spinal cord ischemia is theoretically possible and was observed in one patient in our study. It may be because slow and gradual thrombosis occlusion instead of direct diminishing of the false lumen provides enough time for collateral circulation of intercostal arteries to establish. We tried our best to reserve the left subclavian artery and at least one side of the internal iliac arteries to protect the collateral circulation blood supply of the spinal cord. Another reason may be the small gutter at the landing zone and between parallel stent grafts could provide some blood supply for a period long enough for collateral circulation of the intercostal arteries to establish. Maintaining moderately high blood pressure and sufficient blood volume preoperatively are also important for spinal cord perfusion. Because of small number in our sample, we cannot give a solid conclusion.

The fenestrated stent graft technology includes *in vivo in situ* fenestration and *in vitro* pre-fenestration, and is mainly used to treat aortic arch diseases (33, 34). It can also be used to bridge branch stents on the basis of fenestration to provide blood supply to thoracic and abdominal aortic branch vessels, mostly for distal dissection. The problem with *in situ* fenestrated stent graft technology mainly lies in the changes and covering in the stent during the fenestrating process, which may cause potential damage to the graft and patients, such as ischemia caused by prolonged fenestrated time and iatrogenic complications caused by an improper fenestration operation. Therefore, it is mostly suitable for emergency surgery. Pre-fenestrated stent graft technology mainly includes customizing fenestration from manufacturers after measuring the patient's aortic data and fenestration on the operating table by the physician, but the difficulty lies in the accuracy of stent release, which needs high technical requirements.

In our study, we compared the endoleak rate and branch patency rate of three kinds of EVAR techniques, FSG, BSG, and PSG, in a sample of 291 patients during perioperation. In different case reports, due to differences in sample size, physician technical level, and stent production companies, there exist differences in the endoleak rate and branch patency rate of the three techniques, but the overall trend is consistent with our study (35–38). Based on the scope of application, technical difficulty, short-term and long-term complications, and patient prognosis of the three technologies for aortic pathologies, we consider the parallel stent graft technology to have an endoleak problem that is difficult to solve. There exists technical barriers and durability issues with fenestrated stent graft technology, making it difficult to operate. Branched stent graft technology, as a technology with a wide range of applications, moderate technical difficulty, relatively few complications, and relatively good patient prognosis, can achieve good therapeutic effects and is the future development direction.

## 5 Limitations

The study only focused on evaluating the operational difficulty, surgical cost, and long-term efficacy without considering other important factors such as patient’s quality of life and potential complications associated with each repair method. Therefore, a more comprehensive analysis is needed to fully understand the benefits and drawbacks of each approach. While this study provides some insights into the three EVAR methods, it has several limitations that should be taken into consideration. Further research with larger sample sizes, longer follow-up periods, and comprehensive evaluations is needed to fully understand the efficacy and safety of these repair methods for aortic dissection and aortic aneurysm.

## 6 Conclusions

In conclusion, based on the limited data available, to treat the aortic pathologies involving visceral segment, BSG had a lower endoleak rate and higher branch patency rate when compared with the FSG and PSG techniques. It offers a balance between ease of use and cost. However, it is important to note that further research with larger sample sizes and longer follow-up periods is needed to fully evaluate the efficacy and safety of these repair methods.

## Data Availability

The original contributions presented in the study are included in the article/Supplementary Material, further inquiries can be directed to the corresponding authors.
